# Emotion recognition in relation to tumor characteristics in patients with low-grade glioma

**DOI:** 10.1093/neuonc/noad209

**Published:** 2023-10-31

**Authors:** Femke F Siebenga, Hiska L van der Weide, Floor Gelmers, Sandra E Rakers, Miranda C A Kramer, Anouk van der Hoorn, Roelien H Enting, Ingeborg Bosma, Rob J M Groen, Hanne-Rinck Jeltema, Michiel Wagemakers, Jacoba M Spikman, Anne M Buunk

**Affiliations:** Department of Neurology, Unit of Neuropsychology, University of Groningen, University Medical Center Groningen, Groningen, The Netherlands; Department of Neurology, University of Groningen, University Medical Center Groningen, Groningen, The Netherlands; Department of Radiation Oncology, University of Groningen, University Medical Center Groningen, Groningen, The Netherlands; Department of Neurology, Unit of Neuropsychology, University of Groningen, University Medical Center Groningen, Groningen, The Netherlands; Department of Neurology, University of Groningen, University Medical Center Groningen, Groningen, The Netherlands; Department of Neurology, Unit of Neuropsychology, University of Groningen, University Medical Center Groningen, Groningen, The Netherlands; Department of Neurology, University of Groningen, University Medical Center Groningen, Groningen, The Netherlands; Department of Radiation Oncology, University of Groningen, University Medical Center Groningen, Groningen, The Netherlands; Department of Radiology, University of Groningen, University Medical Center Groningen, Groningen, The Netherlands; Department of Neurology, University of Groningen, University Medical Center Groningen, Groningen, The Netherlands; Department of Neurology, University of Groningen, University Medical Center Groningen, Groningen, The Netherlands; Department of Neurosurgery, University Medical Center Groningen, University of Groningen; Department of Neurosurgery, Faculty of Medicine Universitas Airlangga, Dr. Soetomo General Academic Hospital, Surabaya, Indonesia; Department of Neurosurgery, University Medical Center Groningen, University of Groningen; Department of Neurosurgery, University Medical Center Groningen, University of Groningen; Department of Neurology, Unit of Neuropsychology, University of Groningen, University Medical Center Groningen, Groningen, The Netherlands; Department of Neurology, University of Groningen, University Medical Center Groningen, Groningen, The Netherlands; Department of Neurology, Unit of Neuropsychology, University of Groningen, University Medical Center Groningen, Groningen, The Netherlands

**Keywords:** emotion recognition, frontal tumor location | low-grade glioma, tumor volume

## Abstract

**Background:**

Patients with low-grade gliomas (LGG) treated with surgery, generally function well and have a favorable prognosis. However, LGG can affect neurocognitive functioning. To date, little is known about social cognition (SC) in these patients, although impaired SC is related to social-behavioral problems and poor societal participation. Frontal brain areas are important for SC and LGG frequently have a frontal location. Therefore, the aim of the present study was to investigate whether emotion recognition, a key component of SC, was impaired, and related to general cognition, tumor location, laterality, tumor volume, and histopathological characteristics in patients with LGG, postsurgery, and before start of adjuvant therapy.

**Methods:**

A total of 121 patients with LGG were matched with 169 healthy controls (HC). Tumor location [including (frontal) subregions; insula, anterior cingulate cortex, lateral prefrontal cortex (LPFC), orbitofrontal-ventromedial PFC] and tumor volume were determined on MRI scans. Emotion recognition was measured with the Ekman 60 faces test of the Facial Expressions of Emotion-Stimuli and Tests (FEEST).

**Results:**

Patients with LGG performed significantly lower on the FEEST than HC, with 33.1% showing impairment compared to norm data. Emotion recognition was not significantly correlated to frontal tumor location, laterality, and histopathological characteristics, and significantly but weakly with general cognition and tumor volume.

**Conclusions:**

Emotion recognition is impaired in patients with LGG but not (strongly) related to specific tumor characteristics or general cognition. Hence, measuring SC with individual neuropsychological assessment of these patients is crucial, irrespective of tumor characteristics, to inform clinicians about possible impairments, and consequently offer appropriate care.

**Key Points**

- Emotion recognition is impaired in patients with LGG compared to healthy controls.
- No specific tumor characteristics were associated with emotion recognition in patients with LGG.
- Individual NPA is important to investigate emotion recognition in *all* patients with LGG.

**Importance of the Study**

This is the first study to examine emotion recognition, an important aspect of social cognition, in a large homogeneous group of 121 patients with LGG, postsurgery, and before start of radiotherapy. Patients with LGG performed significantly lower on emotion recognition than healthy controls, and 33.1% showed an impairment compared to norm data. However, we could not identify specific (tumor) characteristics associated with emotion recognition in patients with LGG, namely, general cognition, frontal tumor location (including (frontal) brain subregions), laterality, tumor volume, and histopathological characteristics. Thus, this work addresses the importance of individual neuropsychological assessment in *all* patients with LGG, to inform them timely about possible impairments in emotion recognition. The investigation of impairments in emotion recognition can give rise to appropriate (neuro)psychological treatment selection in patients with LGG.

Low-grade gliomas (LGG) are a diverse group of slow-growing primary brain tumors, predominantly located in the frontal lobe.^1,2^ Patients with LGG are often young to middle-aged adults and have a median survival of approximately more than 10 years after treatment with surgery followed by radiotherapy and chemotherapy.^3,4^ Therefore, it is important to focus on quality of life of which neurocognition is an important aspect. Research shows that mainly the tumor itself, rather than surgical procedures, can have a negative impact on neurocognitive functions, such as attention, memory, language, and executive functions in patients with brain tumors.^5,6^

The neurocognitive function social cognition has barely been investigated in patients with LGG. Social cognitive functions allow the processing of complex social information, with as important elements the ability to recognize other people’s emotional expressions, and to understand others’ thoughts and feelings. Inaccurate recognition or misinterpretation of emotional facial expressions can lead to impairments in social functioning, that is, poor social communication and inappropriate interpersonal behavior.^7–9^ To date, only 2 studies, with relatively small sample sizes, focused on emotion recognition in patients with LGG. Campanella et al. ^10^ found intact emotion recognition in 21 LGG patients preoperatively, with deficits postsurgery that recovered within 4 months to premorbid level, whereas Buunk et al. ^11^ found indications for impairments in emotion recognition in 30 LGG patients, presurgery, that did not worsen postsurgery. Thus, research about emotion recognition after resection of an LGG is limited and results until now are inconclusive.

Measuring social cognition is not easy and tests are often multifactorial, possibly tapping into other general cognitive functions. Given this potential interplay, it is imperative to account for general cognition when examining social cognition. For instance, measuring emotion recognition is usually done by presenting photographs of faces with emotional expressions that have to be recognized. Recognizing these emotional expressions that are only briefly displayed, could also require processing speed or attention. Because patients with glioma show cognitive deficits in several neurocognitive functions,^5,6^ it is important to also take the relationship between general cognition and performance on emotion recognition into account.

Since social cognition is subserved by a fronto-subcortical network, the question arises whether impairments in emotion recognition are related to tumor location, specifically a frontal location.^8,12^ However, the effect of tumor location on cognitive functioning in patients with brain tumors remains debatable^13–16^ and specifically in patients with LGG, the relationship between (frontal) tumor location and social cognition has barely been investigated. Solely one study investigated whether the presence of impairments in emotion recognition, in 30 patients with LGG, was related to frontal tumor location, finding no evidence for such a relationship.^11^ The role of specific frontal regions in emotion recognition was not investigated; merely a crude distinction between frontal and nonfrontal tumors was made in this study. Different regions within the frontal lobe have been associated with emotion processing in healthy individuals, for example, the insula is associated with the recognition of disgust and angry expressions, whereas the anterior cingulate cortex (ACC) is related to processing happy expressions.^17^ Given the limited evidence, it is relevant to investigate whether impaired emotion recognition might be related to damage in specific frontal brain subregions more thoroughly in a large, homogeneous group of patients with LGG. Furthermore, it remains unclear if there is a lateralization effect of emotion recognition in patients with LGG. Research shows that impairments in emotion recognition were more severe in stroke patients with right-sided lesions compared to those with left-sided lesions.^18^ Therefore, it is interesting to investigate if this lateralization effect of emotion recognition also exists in patients with LGG, as this has not yet been investigated.

Impairments in emotion recognition may not only be related to tumor location but also to tumor volume or histopathological subtype in patients with LGG. LGG are diffuse, slow-growing tumors, that can induce reorganization of widely distributed areas in the brain over the years.^19^ Larger tumor volume may involve more distributed networks in the brain, exerting pressure on brain areas concerning these networks, possibly causing impairments in emotion recognition. In addition, histopathological characteristics such as tumor subtype have been associated with cognitive impairments in patients with brain tumors.^20,21^ However, to date, the relationship between emotion recognition and histopathological subtypes has not yet been investigated in patients with LGG.

The aim of the present study was 2-fold: (1) to investigate whether patients with LGG show impairments in emotion recognition, a major aspect of social cognition; (2) to investigate the relationship between emotion recognition and both general cognition and different tumor characteristics in patients with LGG; namely frontal tumor location (including frontal subregions), laterality, tumor volume, and histopathological characteristics, after surgery and before the start of adjuvant therapy.

## Materials and Methods

### Participants

All patients with diffuse mutated isocitrate dehydrogenase (IDH) 1p/19 codeleted oligodendroglioma and diffuse IDH-mutated astrocytoma that were evaluated after surgery and before adjuvant therapy, between November 2017 and April 2022, at the University Medical Center Groningen (UMCG), were eligible for this study. In the Netherlands LGG patients that meet the selection criteria are preferably treated with proton therapy at the on-site Proton Therapy Center in Groningen.^22^ Proton therapy is a new emerging radiotherapy modality that allows to reduce radiation exposure of the surrounding healthy brain tissue, which is particularly of interest for LGG patients with a relatively longer life expectancy.^22^ All patients with LGG eligible for proton therapy are included in a prospective monitoring program that includes a neuropsychological examination before and every 2.5 years after start of proton therapy. The current study only focuses on data collected preradiotherapy, as a baseline measurement. Exclusion criteria were age younger than 18 years, insufficient command of the Dutch language, previous chemotherapy or radiotherapy, neurological or severe psychiatric disorders, and alcohol or drug abuse. A group of healthy controls, matched on sex, age, and educational level, was included, and collected from a database of studies of the UMCG, subdepartment Neuropsychology. Data obtainment occurred in compliance with the ethical regulations of the Medical Ethical Committee of the UMGG. All participants gave written informed consent at the Department of Radiotherapy, in regard to the whole form of care concerning prospective data registration, including consent for collecting clinical and neuropsychological data from medical records.

### MRI Imaging and Radiological Evaluation

All patients received a preradiotherapy MRI scan as part of the radiotherapy preparation including a 3D fluid-attenuation inversion recovery (1.5T Siemens Avanto; TR 5000 ms; TE 335 ms; TI 1800 ms; flip angle 120°, number of averages 1; voxel size 1 × 1 × 1 mm) or were scanned with a similar sequence on a 1.5 or 3T scanner. Tumor locations were scored on the preradiotherapy planning MRI scan by the presence of tumor infiltration in the frontal lobe and 4 specific brain regions (within the frontal lobe): the insula, ACC, lateral prefrontal cortex (LPFC), and orbitofrontal-ventromedial PFC (OFC-vmPFC). The anatomical boundaries of these brain regions were used from MRIcron (v1.0.20190902); the template Ch2better was used with the template of the Brodmann as an overlay. Since the boundaries of tumor infiltration in the brain regions are not circumscript, infiltrations were scored as no infiltration, marginal/limited infiltration (<10% involvement of the specific brain region), or substantial infiltration (>10% involvement of the specific brain region). When a specific region was (partially) resected, the region was scored as having tumor involvement. Tumor location was scored separately by 2 radiation oncologists dedicated to neuro-oncology [HvdW and MK], blinded to each others’ ratings. Subsequently, all discrepancies were solved by consensus during a discussion. The gross tumor volume (GTV) on the preradiotherapy MRI scan was used as a surrogate for tumor volume. The GTV was defined as the postsurgical tumorbed, including the resection cavity, and the residual T2/FLAIR hyperintense zone.

### Neuropsychological Assessment

Patients underwent a neuropsychological assessment (NPA) as part of routine clinical care at the UMCG. This NPA was scheduled prior to start of radiotherapy or within a week after start of treatment. Raw scores were calculated for each patient and adjusted for age, sex, and education (leading to norm scores, in this case percentile scores). Educational level was scored according to the Dutch classification system of Verhage (1964),^23^ ranging from 1 (no primary school) to 7 (university level).

#### Emotion recognition.—

The Ekman 60 faces test of the Facial Expressions of Emotion Stimuli and Tests (FEEST)^24^ was used to measure recognition of facial expressions of emotion, an important aspect of social cognition. Participants are shown 60 static photos, each showing one of the 6 basic emotions, that is, anger, disgust, fear, happiness, sadness, and surprise. The stimuli are presented for 5 seconds, then the participant is asked to choose which emotion label best describes the emotion shown. The total scores range from 0 to 60; for each of the 6 emotions scores range from 0 to 10.

#### General cognition.—

Attention and executive control were assessed by using the Trail Making Test (TMT)^25^ In condition A, the participant is instructed to connect numbered circles in sequential order as quickly as possible. In condition B of the TMT, the participant alternately has to connect numbers and letters in ascending sequence. Scores are the time in seconds needed to complete the conditions.

Encoding and retrieval of verbal information were measured with the 15 Words Test Immediate Recall (15WT IR), the Dutch version of the Auditory Verbal Learning Test.^26^ The participant is presented with 5 presentations of a 15-word list of one word per second, after which the participant is asked to recall as many words as possible. After a 20 min delay, the participant is asked to recall as many words as possible from the presentations of 15WT IR, which is called the 15WT delayed recall (15WT DR). Total scores are the total number of correct recalled words.

### Statistical Analysis

The analyses were conducted in Statistical Package for the Social Sciences (SPSS), Version 28.0. Neuropsychological test data were checked for normal distribution by using quantile–quantile (Q–Q) plots. Parametric statistical tests were used for normally distributed data, otherwise a nonparametric alternative was applied. Differences in demographic characteristics, including gender, age, and educational level between healthy controls and patients with LGG were analyzed using Chi-square and independent *t*-tests. Compared to norm data used in clinical practice, performances below the tenth percentile on the FEEST were considered to be impaired.^27^ To analyze differences in raw scores on the FEEST between patients with LGG and healthy controls, independent *t*-tests were performed. Subsequently, one-sample *t*-tests were used to compare standardized scores on tests for general cognition to a normative sample with a mean of 50 and a standard deviation of 10. Spearman correlations between percentile scores on the FEEST and tests for general cognition were calculated to examine the relationship between emotion recognition and general cognition. Independent *t*-tests were used to compare mean percentile scores on the FEEST between the subgroups of frontal and nonfrontal tumors. Subsequently, independent *t*-tests were used to examine the relationship between emotion recognition and specific (frontal) brain regions; mean percentile scores on the FEEST of patients with substantial tumor infiltration (>10%) and without/marginal tumor infiltration (<10%) in specific (frontal) brain regions, that is, the insula, ACC, LPFC, and OFC-vmPFC, were compared. Additionally, the relationship between emotion recognition and the site of the tumor was examined by comparing mean percentile scores on the FEEST for tumors located in the right versus the left hemisphere, using independent *t*-tests. Patients with midline tumors were excluded from the analysis of lateralization. Spearman’s correlations were performed to calculate associations between percentile scores on the FEEST and tumor volume (GTV). Subsequently, Mann–Whitney *U* tests were used to examine differences in percentile scores between patients with large (GTV > 100 cc) versus small (GTV < 100 cc) tumors. Considering histopathological characteristics, independent *t*-tests were used to compare mean percentile scores on the FEEST between patients with diffuse astrocytoma (IDH-mutant) and diffuse oligodendroglioma (IDH-mutant, 1p/19q-codeleted) were examined. Effect sizes (Cohen’s *d*) were calculated for all between-group comparisons. The overall alpha level (*P*) was set at 0.05. In the case of multiple comparisons Bonferroni–Holm corrections were applied.^28^

## Results

A total of 121 patients with LGG were included for analysis (Table 1). No significant differences between patients with LGG and the 169 healthy controls were found in male–female ratio (*χ* = 1.73, *P* = 0.188), age (*t* = 1.76, *P* = 0.080), and educational level (*χ* = 9.29, *P* = 0.098). A total of 96.7% of patients underwent an NPA before or within the first week after start of radiotherapy and 3.3% had an NPA between 9 days and 15 days after start of radiotherapy.

**Table 1.** Sociodemographic and Clinical Characteristics of Patients with LGG

Characteristic	LGG (*n* = 121)	HC (*n* = 169)
Sex, number of women (%)	60 (49.6)	97 (57.4)
Age in years, mean (SD)	42.5 (12.3)	45.1 (12.9)
Educational level, mean (SD)	5.1 (1.0)	5.3 (1.0)
Histopathology^a^		
Diffuse oligodendroglioma, IDH-mutant, 1p/19q codeleted, *n* (%)	61 (50.4)	
Diffuse astrocytoma, IDH mutated, *n* (%)	60 (49.6)	
WHO tumor grade^b^		
Grade 2, *n* (%)	93 (76.9)	
Grade 3, *n* (%)	28 (23.1)	
Tumor location^c^		
Frontal, *n* (%)	79 (65.3)	
Temporal, *n* (%)	15 (12.4)	
Parietal, *n* (%)	17 (14.0)	
Insular, *n* (%)	7 (5.8)	
Occipital, *n* (%)	2 (1.7)	
Thalamus/brainstem, *n* (%)	1 (0.8)	
Lateralization^d^		
Left-sided, *n* (%)	64 (52.9)	
Right-sided, *n* (%)	56 (46.3)	
Midline, *n* (%)	1 (0.8)	
GTV in cc, mean (SD)	58.5 (54.2)	
Time interval between last surgery and NPA in weeks, median (range)	11.0 (4–335)	
Time interval between last surgery and start RT in weeks, median (range)	10.0 (5–335)	
Wait and scan policy >6 months since diagnosis, *n* (%)	46 (38.0)	
Type of last surgery, *n* (%)		
Biopsy	8 (6.6)	
Craniotomy under general anesthesia	70 (57.9)	
Advanced craniotomy awake	43 (35.5)	
Extent of last tumor resection		
<25 %, *n* (%)	9 (7.4)	
25–90%, *n* (%)	74 (61.2)	
>90%, *n* (%)	38 (31.4)	
Use of steroids, *n* (%)	2 (1.7)	
Active focal epileptic symptoms within 2 weeks before NPA, *n* (%)	12 (9.9)	
Use of anti-epileptic treatment		
Mono therapy, *n* (%)	61 (50.4)	
Poly therapy, *n* (%)	21 (17.4)	
None, *n* (%)	39 (32.2)	

*Notes:* LGG = low-grade glioma; HC = healthy controls; educational level = 7-point scale ranging from 1 (no primary school) to 7 (university) according to Verhage (1964); RT = radiotherapy; IDH = isocitrate dehydrogenase; GTV = gross tumor volume; NPA = neuropsychological assessment.

^a^According to WHO 2021 classification.

^b^According to WHO 2016 classification.

^c^Indicated as the location of the main bulk of tumor.

^d^Indicated as lateralization of the main bulk of tumor.

### Emotion Recognition in Patients With LGG Compared to Healthy Controls

Patients with LGG showed significantly lower performances on the FEEST total and the subscales “Anger,” “Disgust,” “Fear” and “Sadness,” compared to healthy controls, with moderate to large effect sizes (Table 2). Furthermore, 33.1% of patients showed an impaired performance on the FEEST compared to norm scores, that is, a performance below the tenth percentile.

**Table 2.** Performance on Emotion Recognition Compared to Healthy Controls

	LGG (*n* = 121)	HC (*n* = 169)			
	M (SD)	M (SD)	*t*	*P*	*d* ^a^
Emotion recognition					
Anger	7.6 (1.7)	8.2 (1.6)	3.06	0.002^*^	0.36
Disgust	6.7 (2.2)	8.1 (1.9)	6.02	<0.001^*^	0.74
Fear	5.9 (2.4)	6.8 (2.3)	3.34	<0.001^*^	0.40
Happiness	9.9 (0.4)	9.9 (0.3)	1.52	0.129	0.18
Sadness	6.3 (2.1)	7.4 (1.7)	4.85	<0.001^*^	0.60
Surprise	8.9 (1.2)	9.0 (1.2)	0.85	0.395	0.10
Total	45.2 (6.6)	49.5 (5.3)	5.98	<0.001^*^	0.74

*Note:* LGG = low-grade glioma; HC = healthy controls.

^*^Significant *P*-value < Bonferroni–Holm corrected alpha.

^a^Cohen’s *d,* effect size.

### General Cognition and Emotion Recognition

Impairments in general cognition varied from 11.7% to 28.3% of patients with LGG. However, patients with LGG solely showed a significantly lower score on the 15WT-IR than the normative group (Table 3). Furthermore, the relationship between emotion recognition and general cognition was examined: significant and small correlations were found between total percentile scores of the FEEST and percentile scores of the TMT-A, TMT-B, and 15WT-IR (*r* = 0.22–0.29) (Table 3).

**Table 3.** General Cognition in Patients with LGG

	M (SD)	*t*	*P*	*d* ^a^
Psychomotor speed and executive control				
TMT—A (*n* = 121)	45.8 (32.6)	−1.410	0.161	0.13
TMT—B (*n* = 120)	44.5 (31.0)	−1.936	0.055	0.18
Verbal memory (encoding and retrieval)				
15WT IR (*n* = 120)	34.1 (29.1)	−5.98	<0.001^*^	0.55
15WT DR (*n* = 120)	46.4 (29.1)	−1.371	0.173	0.13

*Note:* Mean percentile scores are presented.

LGG = low-grade glioma; TMT = trail making test; 15WT IR = 15 words test immediate recall; 15WT DR = 15 words test delayed recall.

^*^Significant *P*-value < Bonferroni–Holm corrected alpha.

^a^Cohen’s *d,* effect size.

### Tumor Location

Table 4 shows performances on the FEEST of patients with frontal and nonfrontal tumors; no statistically significant differences were found between groups. Furthermore, the mean percentile scores on the FEEST of patients with tumors with and without tumor infiltration in 4 specific (frontal) subregions, that is, the insula, ACC, LPFC, and OFC-vmPFC, showed no significant differences for patients with and without infiltration of these brain regions (Table 5). In addition, no significant differences were found in mean percentile scores on the FEEST between patients with tumors located in the left versus the right hemisphere (Table 6). Furthermore, differences between left- and right-hemispheric tumors were examined within the group of frontal tumors; likewise, no significant differences were found.

**Table 4.** Emotion Recognition in Patients with Frontal Versus Nonfrontal Tumors

	Frontal (*n* = 79)		Nonfrontal (*n* = 42)		t	P	*d* ^a^
	RS	PS	RS	PS			
Emotion recognition	M (SD)	M (SD)	M (SD)	M (SD)			
Anger	7.4 (1.8)	47.0 (31.5)	7.9 (1.6)	53.6 (31.4)	−1.10	0.274	0.21
Disgust	6.6 (2.3)	40.3 (31.4)	6.8 (2.1)	39.0 (29.5)	0.17	0.863	0.03
Fear	6.1 (2.4)	50.6 (28.8)	5.4 (2.3)	40.9 (28.9)	1.76	0.080	0.34
Happiness	9.8 (0.4)	86.6 (32.0)	9.9 (0.3)	89.1 (30.0)	−0.42	0.676	0.08
Sadness	6.4 (2.0)	42.4 (29.6)	6.1 (2.4)	38.2 (32.6)	0.73	0.466	0.14
Surprise	8.8 (1.2)	62.0 (33.8)	8.9 (1.4)	66.6 (36.3)	−0.68	0.496	0.13
Total	45.3 (6.5)	31.9 (28.5)	45.0 (6.9)	29.4 (28.1)	0.45	0.651	0.09

*Notes:* RS = raw score, PS = percentile score; FEEST = Facial Expression of Emotion Stimuli and Test.

Independent *t*-tests were used to compare percentile scores on the FEEST.

^a^Cohen’s *d,* effect size.

^*^Significant *P*-value < Bonferroni–Holm corrected alpha.

**Table 5.** Emotion Recognition in Patients With and Without Infiltration of (Frontal) Subregions

	Insula		ACC		LPFC		OFC-vmPFC	
	Yes (*n* = 21)	No (*n* = 58)	Yes (*n* = 35)	No (*n* = 44)	Yes (*n* = 40)	No (*n* = 39)	Yes (*n* = 22)	No (*n* = 54)
Emotion recognition	M (SD)	M (SD)	M (SD)	M (SD)	M (SD)	M (SD)	M (SD)	M (SD)
Anger	54.9 (30.3)	44.2 (31.7)	44.7 (27.4)	48.9 (34.6)	44.2 (32.0)	50.0 (31.1)	53.5 (28.0)	44.1 (32.8)
Disgust	33.1 (25.9)	42.5 (33.0)	31.5 (26.6)	46.8 (33.5)	33.0 (27.3)	47.3 (34.0)	36.8 (29.6)	41.5 (32.4)
Fear	41.3 (30.3)	54.0 (27.8)	46.8 (31.1)	53.7 (26.9)	42.7 (29.6)	58.7 (26.0)	51.2 (33.1)	50.4 (27.0)
Happy	84.0 (34.2)	87.5 (31.5)	85.5 (32.5)	87.4 (32.1)	85.0 (33.2)	88.2 (31.3)	86.7 (31.5)	86.5 (32.6)
Sadness	49.0 (30.2)	40.0 (29.3)	40.8 (29.8)	43.8 (29.7)	36.2 (26.0)	48.9 (31.9)	49.1 (29.9)	39.4 (29.2)
Surprise	61.1 (36.5)	62.4 (33.1)	66.9 (33.5)	58.1 (33.9)	59.0 (35.1)	65.2 (32.5)	56.8 (32.1)	64.4 (34.6)
Total	31.0 (31.4)	32.2 (27.7)	28.9 (28.9)	34.2 (28.3)	23.7 (27.0)	40.3 (27.8)	33.4 (30.4)	31.2 (27.8)

*Note:* Tumor location is indicated as >10% tumor infiltration in a specific brain region within the frontal lobe.

FEEST = Facial Expressions of Emotion Stimuli and Tests; ACC = anterior cingulate cortex; LPFC = lateral prefrontal cortex; OFC-vmPFC = orbitofrontal cortex ventromedial prefrontal cortex.

Independent *t*-tests were used to compare percentile scores on the FEEST between patients with and without infiltration the Insula, ACC, LPFC, and OFC-vmPFC.

^*^Significant *P*-value < Bonferroni–Holm corrected alpha.

**Table 6.** Emotion Recognition in Patients with Left- and Right-Sided Tumors

	Total (*n* = 121)				Frontal tumors (*n* = 79)			
	Left (*n* = 64)	Right (*n* = 56)			Left (*n* = 41)	Right (*n* = 38)		
Emotion recognition	M (SD)	M (SD)	*t*	*P*	M (SD)	M (SD)	*t*	*P*
Anger	44.9 (30.7)	53.5 (31.6)	−1.51	0.134	44.2 (31.9)	50.1 (31.2)	−0.83	0.408
Disgust	38.4 (29.8)	41.9 (31.6)	−0.63	0.532	38.5 (32.2)	41.7 (30.9)	−0.45	0.652
Fear	49.3 (24.9)	45.7 (33.1)	0.67	0.503	52.7 (24.8)	48.4 (32.8)	0.66	0.517
Happiness	86.4 (32.0)	88.4 (31.0)	−0.35	0.731	85.5 (32.7)	87.8 (31.8)	−0.32	0.750
Sadness	43.3 (28.0)	38.8 (33.4)	0.78	0.437	43.8 (27.9)	41.0 (31.6)	0.43	0.668
Surprise	58.2 (32.8)	69.0 (36.0)	−1.73	0.087	56.4 (32.7)	68.1 (34.3)	−1.55	0.063
Total	29.8 (26.7)	33.0 (30.2)	−0.61	0.543	31.2 (28.7)	32.6 (28.6)	−0.22	0.825

*Note:* FEEST = Facial Expressions of Emotion Stimuli and Tests.

Independent *t*-tests were used to compare percentile scores on the FEEST.

^*^Significant *P*-value < Bonferroni–Holm corrected alpha.

### Tumor Volume and Histopathological Characteristics

A significant negative, small correlation coefficient was found between GTV and the total percentile score on the FEEST (*r* = −0.19, *P* = 0.041), indicating patients with larger tumors score worse on emotion recognition. However, a comparison between patients with large (GTV > 100 cc) and small (GTV < 100 cc) tumors revealed no significant differences in FEEST percentile scores (*P* > 0.05). Additionally, within the group of frontal tumors, we found no relationship between GTV and the total percentile score on the FEEST (*r* = −0.11, *P* = 0.339). Furthermore, no significant differences in total percentile scores on the FEEST between patients with diffuse astrocytoma (IDH-mutant) and diffuse oligodendroglioma (IDH-mutant, 1p/19q-codeleted; *t* = -0.04, *P* = 0.969) were found.

## Discussion

The present study showed that one-third of the patients with LGG, after surgery and before adjuvant therapy, have impairments in emotion recognition, compared to healthy controls. We deem it not likely that surgery caused these impairments in emotion recognition in our patient group, given previous studies that showed that surgery does not have additional negative effects on cognition, including social cognition.^6,10,11^ Hence, we presume that these impairments are caused by the tumor itself. Overall, this study did not yield convincing evidence for a relationship between impaired emotion recognition and specific tumor characteristics, namely, frontal tumor location, laterality, tumor volume, and histopathological characteristics.

This is the first study investigating impairments in emotion recognition in a large homogeneous group of patients with LGG after surgery but before treatment with radiotherapy and/or chemotherapy. Compared to healthy controls, patients with LGG showed worse emotion recognition, specifically impaired recognition of negatively valenced emotions, namely, anger, disgust, fear, and sadness. Our results add to previous preliminary evidence showing that a substantial proportion of patients with LGG suffer from impairments in emotion recognition postoperatively and perform significantly worse than healthy controls.^10,11^ Considering the separate emotions, happiness is most easily recognized by both LGG patients and healthy controls, consistent with prior research in healthy controls and patients with traumatic brain injury (TBI),^29,30^ showing that positively valenced emotions are recognized more accurately. Thus, this distinction is not unique for patients with LGG, and may arise from happiness being the sole positive emotion in the FEEST, making it more distinguishable due to the distinctive feature of a smile. Interestingly, patients with LGG recognized negative emotions even worse compared to healthy controls, with the severity of deficits varying among discrete negative emotions, indicating that this was due to brain pathology and not to difficulty alone. As previous studies showed that impaired recognition of negative emotions is related to behavioral problems in patients with TBI (eg, aggression problems due to an inability to adequately adjust behavior in response to others),^31,32^ measuring social cognition with a thorough neuropsychological examination in clinical practice is important. The relation between impaired emotion recognition impairments and behavioral problems in LGG patients has not been investigated yet. Knowledge of whether there is such a relationship in this patient group is highly relevant to inform patients timely about possible consequences for daily functioning and social relationships.

Considering the possible interplay between emotion recognition and general cognition, general cognitive functions, such as memory, attention, and executive control, were assessed and scores on these variables were correlated to emotion recognition. Patients performed on most measures at the same level as the normative sample, except verbal encoding, where performance was significantly lower, and hence, impaired. Furthermore, significant though weak correlations were found between emotion recognition and 3 measures for general cognition, that is, TMT-A, TMT-B, and 15WT-IR. We do not consider it likely that these relationships are causal but deem it plausible that lower scores on these different measures are underlined by a general process of reduced brain function, affecting different cognitive processes simultaneously.

Despite the presumed important role of frontal areas and the right hemisphere in emotion recognition, the present study did not show a significant relation between emotion recognition and frontal tumor location, specific (frontal) brain subregions (insula, ACC, LPFC, and OFC-vmPFC), and laterality in patients with LGG. The current study examined the relationship between emotion recognition and tumor location by detecting structural brain damage with MRI, allowing us to identify brain areas possibly associated with impaired emotion recognition, as this had not yet been investigated thoroughly in a large sample of patients with LGG. In other patient groups, such as patients with acute brain damage due to TBI or stroke, the relationship between specific brain areas and emotion recognition has been investigated, and in these groups, associations of impaired emotion recognition with specific locations, such as frontal lesions^33,34^ and right-hemispheric lesions^18^ have been found. However, patients in the current study differ significantly from patient populations with acute brain damage, as LGG is characterized by a slow and gradual growth over time. Since LGG are slow-growing tumors, this slow invasion of brain tissue allows to shift the functions underlined by this tissue as part of brain networks to other areas. This ability of neural networks to adapt to damage is called postlesional plasticity.^35^ Consequently, the originally infiltrated areas may not be significant to the neural network responsible for recognizing specific emotions anymore, and relevant hubs in these networks may be located differently across patients. This diversity may explain the lack of significant findings regarding a relation between a specific brain area and a brain function, such as emotion recognition. If postlesional plasticity plays a role, this may also imply that impairment of emotion recognition is less severe in patients with LGG than in patients with sustained acute injuries, such as TBI. Although to date there are no studies that have directly compared these groups, some indirect evidence to suggest this possibility is supported in a meta-analysis of facial affect recognition impairments after moderate to severe traumatic brain injury.^36^ The authors analyzed 13 studies comparing adults with TBI to matched healthy controls. They calculated a mean Hedges unbiased effect size (comparable to Cohen’s *d*) with an absolute value of 1.11, which is substantially larger than the effect size we found in our study (0.74). Also, we found that 33% of our patients performed below the 10th percentile (comparable to an SD of −1.3). Babbage *et al*. ^36^ created a model to calculate the proportion of people below different cutoffs and found that 50%–41% would perform below cutoffs between 1.0 and 1.5 SD. Another plausible explanation for the lack of findings is that investigating structural brain damage with MRI might not be sensitive enough to detect associations between emotion recognition and tumor location. It is known that social cognition in patients with brain tumors (right-sided diffuse LGG or mixed groups with high- and low-grade glioma) is underpinned by connectivity among networks enabling mentalizing or emotion recognition, that is, the inferior frontal fasciculus (IFOF) and the superior longitudinal fasciculus appear to be important, based on voxel-based symptom lesion mapping (VLSM) or electrostimulation mapping.^37–39^ Therefore, the initial investigation of the current study requires further research to also identify possible neural underpinnings of emotion recognition in patients with LGG with more advanced brain imaging techniques, and in particular, looking at the role of connectivity in networks. Because the majority of patients have a tumor located in the frontal lobe, examination of underlying frontal neural networks in relation to impaired emotion recognition is specifically of interest.

Regarding tumor volume, a significant but very weak relationship was found between emotion recognition and tumor volume in patients with LGG. However, 65% of the tumors of patients in our study were localized in the frontal lobe; when we investigated this relationship between emotion recognition and tumor volume in this group only, it was not significant anymore. In addition, no differences in emotion recognition between patients with large (GTV > 100 cc) and small (GTV < 100 cc) tumors were found. So, overall, there is no convincing evidence for a relation between tumor volume and impaired emotion recognition in patients with LGG. Furthermore, histopathological subtype was not related to a lower ability to recognize emotions in patients with LGG. Hence, tumor subtype cannot be seen as a decisive factor that determines the extent to which emotion recognition is impaired; we were the first to investigate this in patients with LGG.

Some limitations of our study have to be taken into account. First, patients did not all have the same previous surgical procedures, as 8 patients only underwent a biopsy and the other 113 patients underwent craniotomy under general anesthesia or advanced awake craniotomy. However, previous studies show the LGG itself, rather than resection, can cause cognitive impairments.^6,10,11^ Therefore, we assume that biopsy, a less invasive surgery procedure than total resection, will have negligible effects on cognition. Second, tumor volume was defined as GTV after surgery, an important measure to map the tumor area to be irradiated, since preoperative tumor volume data was not available. Because GTV was used as a surrogate for tumor volume, future research could look at the effect of presurgery determined tumor volume on emotion recognition. Lastly, the aim of the present study was to examine emotion recognition in patients with LGG, after surgery, but before start of radiotherapy. However, 3.3% of the patients underwent an NPA between 9 and 15 days after the start of proton therapy, due to planning problems at the beginning phase after proton therapy was initiated in Groningen, the Netherlands. It is expected this would not have influenced the results, as previous studies showed that cognitive functions remained largely stable in the first years in patients with brain tumors treated with radiotherapy.^40,41^

### Conclusions

Taken together, our study shows that postsurgery patients with LGG were impaired on a crucial aspect of social cognition, that is emotion recognition. However, we could not identify specific tumor characteristics, such as tumor location and tumor volume, to be associated with emotion recognition. In addition, impairments in emotion recognition could not be explained by general cognitive impairments. Therefore, individual NPA is of high importance to investigate emotion recognition in *all* patients with LGG. Because LGG patients with impairments in emotion recognition are presumed to be at risk for developing social-behavioral problems and poor social participation, measuring social cognition timely with a thorough neuropsychological examination is crucial. The investigation of the impact of impairments in emotion recognition on various aspects of daily life in patients with LGG can give rise to appropriate (neuro)psychological treatment selection.

## Data Availability

Raw data were generated at the UMCG, subdepartment Neuropsychology. Derived data supporting the findings of this study are available from the corresponding author [FFS] on request.
